# HIMH0021 attenuates ethanol-induced liver injury and steatosis in mice

**DOI:** 10.1371/journal.pone.0185134

**Published:** 2017-11-01

**Authors:** Yongjun Lee, Dong-Joo Kwon, Young Han Kim, Moonjin Ra, Seong Il Heo, Won Gyeong Ahn, Jeong-Ran Park, Seoung Rak Lee, Ki Hyun Kim, Sun Young Kim

**Affiliations:** 1 Hongcheon Institute of Medicinal Herb, 101 Yeonbongri, Hongcheon, Republic of Korea; 2 School of Pharmacy, Sungkyunkwan University, Suwon, Korea; University of Louisville School of Medicine, UNITED STATES

## Abstract

Chronic alcohol consumption causes alcohol-induced lipogenesis and promotes hepatic injury by preventing the oxidation of hepatocellular fatty acids through the suppression of the activation of AMP-activated protein kinase (AMPK). HIMH0021, an active flavonoid compound, which is a component of the *Acer tegmentosum* extract, has been shown to protect against liver damage caused by alcohol consumption. Therefore, in this study, we aimed to determine whether HIMH0021 could regulate alcoholic fatty liver and liver injury in mice. Oral administration of 10 days of Lieber-DeCarli ethanol plus a single binge of 30% ethanol (chronic-plus-binge model) induced steatosis and liver injury and inflammation in mice, which appears similar to the condition observed in human patients with alcohol-related diseases. HIMH0021, which was isolated from the active methanol extract of *A*. *tegmentosum*, inhibited alcohol-induced steatosis and attenuated the serum levels of alanine aminotransferase (ALT) and aspartate aminotransferase (AST) during hepatocellular alcohol metabolism, both of which promote lipogenesis as well as liver inflammation. Treatment with HIMH0021 conferred protection against lipogenesis and liver injury, inhibited the expression of cytochrome P4502E1, and increased serum adiponectin levels in the mice subjected to chronic-plus-binge feeding. Furthermore, in hepatocytes, HIMH0021 activated fatty acid oxidation by activating pAMPK, which comprises pACC and CPT1a. These findings suggested that HIMH0021 could be used to target a TNFα-related pathway for treating patients with alcoholic hepatitis.

## Introduction

Alcohol-related diseases can be divided into two main categories: alcohol dependence and alcohol abuse [1–2]. Alcohol dependence, which is also known as alcoholism, is a common substance-abuse disorder, where the patients are at an increased risk for cirrhosis, gastrointestinal bleeding, pancreatitis, and a wide variety of cancers. Alcohol abuse is the second most common cause of cirrhosis in the United States after hepatitis. Although there are a few known causes of alcohol-related diseases, the mechanisms underlying alcoholic pathogenesis are incompletely unknown. Several risk factors play an important role in the pathogenesis of alcohol-related diseases, including inflammation, hormone, oxidative stress, genetics, and hepatic mechanism [3–5]. The initial stage of alcohol-related diseases involves the development of fatty liver (steatosis) and accumulation of triglycerides in hepatocytes, which is a reversible condition. If alcohol consumption is continued, steatosis can progress to a potentially pathological condition such as steatohepatitis, fibrosis, cirrhosis, and even hepatocellular carcinoma, especially in the presence of other co-morbidity factors [6].

Alcohol exposure can also induce fatty liver by increasing the NADH/NAD+ ratio, increasing sterol regulatory element-binding protein-1 (SREBP-1) activity, decreasing peroxisome proliferator-activated receptor-α (PPAR-α) activity, and increasing complement C3 hepatic levels. Alcohol exposure also increases NADH production in the process of metabolism by cytosolic alcohol dehydrogenase (ADH) and mitochondrial aldehyde dehydrogenase (ALDH2). Several studies have also shown that NADH is implicated in the disruption of many alcohol-related reactions in the cytoplasm and mitochondria, which leads to suppression of energy supply and fatty acid oxidation, which in turn results in alcoholic fatty liver [7–9].

Long-term alcohol exposure can increase the levels of sterol regulatory element-binding protein-1 (SREBP-1), a master transcription factor that regulates lipogenic enzyme expression, by decreasing the activities of AMP-activated protein kinase and sirtuin-1 [10,11]. Upregulation of SREBP-1 activity in fatty liver can result in the production of tumor necrosis factor-α (TNF-α), but TNF-α can also contribute to the development of alcoholic fatty liver by upregulating SREBP activity. Alcohol consumption can also reduce the production of peroxisome proliferator-activated receptor-a (PPARa), a key transcriptional regulator of lipolytic enzymes [12, 13]. PPAR-α agonists can mitigate alcoholic fatty liver by upregulating PPAR-α and insulin signaling pathways while downregulating SREBP-1 activity and suppressing TNF-α production.

AMPK is known to activate the phosphorylation of the target enzymes involved in lipid metabolism in many tissues, thus promoting fatty acid oxidation by inactivation of acetyl-CoA carboxylase (ACC) and activation of malonyl Co-A decarboxylase (MCD). ACC, a rate limiting enzyme for fatty acid biosynthesis, catalyzes the conversion of acetyl-CoA to malonyl-CoA, which is a potent inhibitor of carnitine palmitoyltransferase-1 (CPT-1). CPT-1 plays an important role in the transportation of fatty acids, which are metabolized via the mitochondrial β-oxidation pathway. MCD degrades malonyl-CoA [14]. Subsequently, alcohol-mediated inhibition of AMPK can result in the activation of ACC and inactivation of MCD and then lead to decreased degradation of malonyl-CoA. The increased concentration of cellular malonyl-CoA can inhibit mitochondrial CPT-1 production, leading to decreased transport of fatty acids into the mitochondria and subsequent oxidation. Furthermore, suppression of adiponectin, a known activator of AMPK, significantly induces SREBP-1c expression in the mouse liver [15].

*Acer tegmentosum* extract has been shown to protect against liver injury caused by alcohol consumption [16, 17]. HIMH0021, an active flavonoid compound, is a component of the *A*. *tegmentosum* extract. The pharmacokinetics of HIMH0021 following oral and intravenous administration have been studied previously. The oral pharmacokinetics of HIMH0021 in mice have also been characterized using a short T_max_ and low bioavailability [18]. However, it is not clear whether HIMH0021 also provides protection from liver injury caused due to chronic alcohol consumption.

Given the close association between HIMH0021 and alcoholic metabolism, we hypothesized that HIMH0021 plays a role in regulating alcoholic steatosis and liver injury in vivo.

To test this hypothesis, we generated a dietary-modified functional liver injury mouse model, where the liver injury was induced by ethanol feeding (chronic-binge feeding model). The mouse chronic-binge feeding model exhibits immune responses that underlie alcoholic liver injury, which leads to alcoholic hepatitis [19–21]. The mice were then administered HIMH0021 to determine its role in regulating hepatic lipid accumulation and liver damage in vivo. We then also went on to determine the mechanisms underlying the effects of HIMH0021. Our results indicated that HIMH0021 inhibits alcohol-induced steatosis through the AMPK/cpt1a signaling pathway and protection liver injury from alcohol consumption in vivo, thus suggesting that it might be an effective therapeutic molecule for use in the treatment of alcoholic liver diseases.

## Materials and methods

### Isolation and identification procedures

NMR spectra were recorded on a Varian UNITY INOVA 700 NMR spectrometer operating at 700 MHz (^1^H) and 175 MHz (^13^C), with chemical shifts given in ppm (δ). Preparative high performance liquid chromatography (HPLC) using a Waters 1525 Binary HPLC pump with a Waters 996 Photodiode Array Detector (Waters Corporation, Milford, CT, USA) was also performed. Semi-preparative HPLC was conducted using a Shimadzu Prominence HPLC System with SPD-20A/20AV Series Prominence HPLC UV-Vis Detectors (Shimadzu, Tokyo, Japan). Silica gel 60 (Merck, 70–230 mesh and 230–400 mesh) and RP-C18 silica gel (Merck, 40–63 μm) were used for column chromatography. The packing material for molecular sieve column chromatography was Sephadex LH-20 (Pharmacia, Uppsala, Sweden).

### Mice and ethanol feeding protocols

Male C57BL/6 mice (Dae Han Biolink, Korea) weighing 20–25 g were individually housed at controlled temperatures (23 ± 1°C) with a 12-h light/dark cycle. The mice were allowed at least 1 week to adapt to their environment before the experiments were started. The methods of euthansia using isoflurane for the animal sacrifice and all the protocols for the animal experiments were approved by the Hongcheon Institute of Medicinal Herb Institutional Animal Care and Use Committee (HIMH 15–05). The feeding protocol (the NIAAA model) involved chronic-binge feeding, in which mice were initially fed the control Lieber-DeCarli diet (Bio-Serv, Frenchtown, NJ) ad libitum for 5 days to acclimatize them to a liquid diet and then fed the control or ethanol Lieber-DeCarli diet for 10 days, as prescribed for the chronic-feeding group. On day 11, the ethanol-fed and control mice were gavaged early in the morning with a single dose of ethanol (5 g ethanol/kg of body weight) or isocaloric dextrin-maltose, respectively. The mice were killed 9 h later using isoflurane.

### Body weight and food intake

The mice were weighed daily, and the food intake was measured using a feeding tube between 3 PM and 5 PM.

### Evaluation of biochemical parameters

Nine hours after ethanol administration, the animals were anesthetized using isoflurane, and 500 μL of blood was collected from the orbital sinus by traumatic avulsion of the globe from the orbit using a pair of tissue forceps. The blood was allowed to clot and then centrifuged at 4000 ×*g* for 10 min. The plasma was separated and used for alanine aminotransferase (ALT), aspartate aminotransferase (AST), triglyceride, and cholesterol assays using Kornelab 20XT (Thermo, USA).

### ELISA

Plasma levels of total adiponectin and hepatic TNF-α were determined using ELISA, which was performed using a commercial ELISA kit from R&D Systems (Minneapolis, MN).

### Western blot analysis

Mouse liver homogenates were prepared using a RIPA buffer containing protease and tyrosine phosphatase inhibitor cocktail (Sigma). The protein concentration of the lysates was determined using the Bicinchoninic (BCA) Protein Assay Kit (Thermo Scientific, IL, USA), according to the manufacturer’s instructions. The isolated soluble proteins (20 mg) were separated on 8–15% SDS-polyacrylamide gels. The separated proteins were then electroblotted onto nitrocellulose transfer membranes (Bio-Rad, Hercules, CA, USA). The membranes were incubated with 5% skim milk for 1 h and then probed with the following 1:1,000-diluted antibodies: anti-pAMPKa, anti-AMPKa, anti-pACC, anti-ACC, anti-CYP2E1, anti-CPT1a (Cell Signaling Technology), and anti-β-actin (Santa Cruz Biotechnology) for 18 h at 4°C. After 3 washes for 10 min each, polyclonal anti-rabbit or mouse HRP-conjugated secondary antibodies (Santa Cruz Biotechnology), which were linked to HRP-bound protein complexes and developed with a Pierce ECL Western Blot substrate (Thermo Fisher Scientific), were added to the mixture. Band densitometry was performed using ImageJ (National Institutes of Health, Bethesda, MD, USA) and expressed as fold change relative to β-actin.

### RNA isolation and real-time RT-PCR

Total RNA was extracted from liver tissues using the TRIzol reagent (Invitrogen), as recommended by the manufacturer. Total RNA (1 mg) was then reverse-transcribed and analyzed using real-time PCR with a Dice TP 800 Thermal Cycler and SYBR Premix ExTaq (Takara Bio). The mRNA expression levels were calculated using β-actin as the control. The primer sequences used were as follows: mouse Acc, forward 5′-TGACAGACTGATCGCAGAGAAAG-3′ and reverse 5′-TGGAGAGCCCCACACACA-3′; mouse CPT1a, forward 5′-TGGCATCATCACTGGTGTGTT-3′ and reverse 5′-GTCTAGGGTCCGATTGATCTTTG-3′; mouse Ppar-a, forward 5′-AACATCGAGTGTCGAATATGTGG-3′ and reverse 5′- AGCCGAATAGTTCGCCGAAAG-3′; and mouse β-actin, forward 5′-GGCTGTATTCCCCTCCATCG-3′ and reverse 5′-CCAGTTGGTAACAATGCCATGT-3′.

### Histopathology

The liver tissues were fixed with 10% formalin for 24 h and embedded in paraffin. Tissues were processed and embedded in paraffin using standard procedures. Liver sections (4 μm) were stained with hematoxylin and eosin. Three to five images were acquired for each slide at 200× magnification and transferred to a computer for analysis.

### Immunohistochemistry and oil red O staining

The paraffin-embedded sections (4-μm thick) were placed on slides, dewaxed using xylene, and then dehydrated through an ethanol series with cumulatively increasing concentrations. Next, the sections were incubated in 3% H_2_O_2_, blocked with 10% goat serum and incubated with the anti-CYP2E1 antibody for 1 h at 4°C. The slides were then washed with tris-buffered saline-Tween 20, incubated with an appropriate biotin-conjugated rabbit anti-goat IgG secondary antibody (1:100; Vector Laboratories, Burlingame, CA, USA), and developed with the avidin biotin complex and 3,39-diaminobenzidine detection reagents (Vector Laboratories), which were prepared according to the manufacturer’s instructions. For Oil Red O staining, frozen sections of the liver (5-μm thick) were stained with Oil Red O solution (Sigma) for 10 min, washed, and then counterstained with hematoxylin for 45 s (DAKO, Carpinteriam CA).

### TUNEL assay

DNA fragmentation characteristic of apoptosis was examined using a TdT (terminal deoxynucleotidyl transferase)-FragEL kit (Oncogene Research Products, San Diego, CA). Briefly, 4% paraformaldehyde-fixed tissue samples were embedded in paraffin and 5-μm sections were cut. Replicate sections were rehydrated and permeabilized with proteinase K (20 μg/mL) for 20 min at 25°C. Next, endogenous peroxidases were inactivated by covering the sections with 3% H_2_O_2_ for 5 min. After incubation for 5 min in TdT buffer (200 mM sodium-cacodylate, 30 mM Tris, 0.3 mg/mL BSA, 0.75 mM CoCl2, pH 6.6), the slides were covered with TdT and biotinylated dUTP and incubated for 1.5 h at 37°C in a humidified chamber. Negative controls were incubated with biotinylated dUTP in the TdT buffer without the enzyme. The reaction was terminated by adding the stop buffer (0.5 M EDTA, pH 8.0) for 5 min at RT. After blocking with 4% BSA, the slides were incubated with a streptavidin-horseradish peroxidase conjugate for 30 min. The sections were then incubated in DAB for 12 min at RT and counterstained with methyl green. Apoptotic cells were identified by their staining properties and using morphological criteria (cell shrinkage, chromatin condensation and/or margination, and apoptotic bodies).

### Statistical analysis

All the statistical data were analyzed using GraphPad Prism 5.0 (GraphPad Software, CA, USA). The results were expressed as the mean ± standard deviation (SD). Comparisons between groups were performed using unpaired Student’s t-test. One-way analysis of variance (ANOVA) was used for comparisons between multiple groups. Statistical significance was considered at P < 0.05. Mean values appeared to be normally distributed, with each figure documenting an appropriate statistical test.

## Results

### Active compound isolation and identification

**Fig 1:**
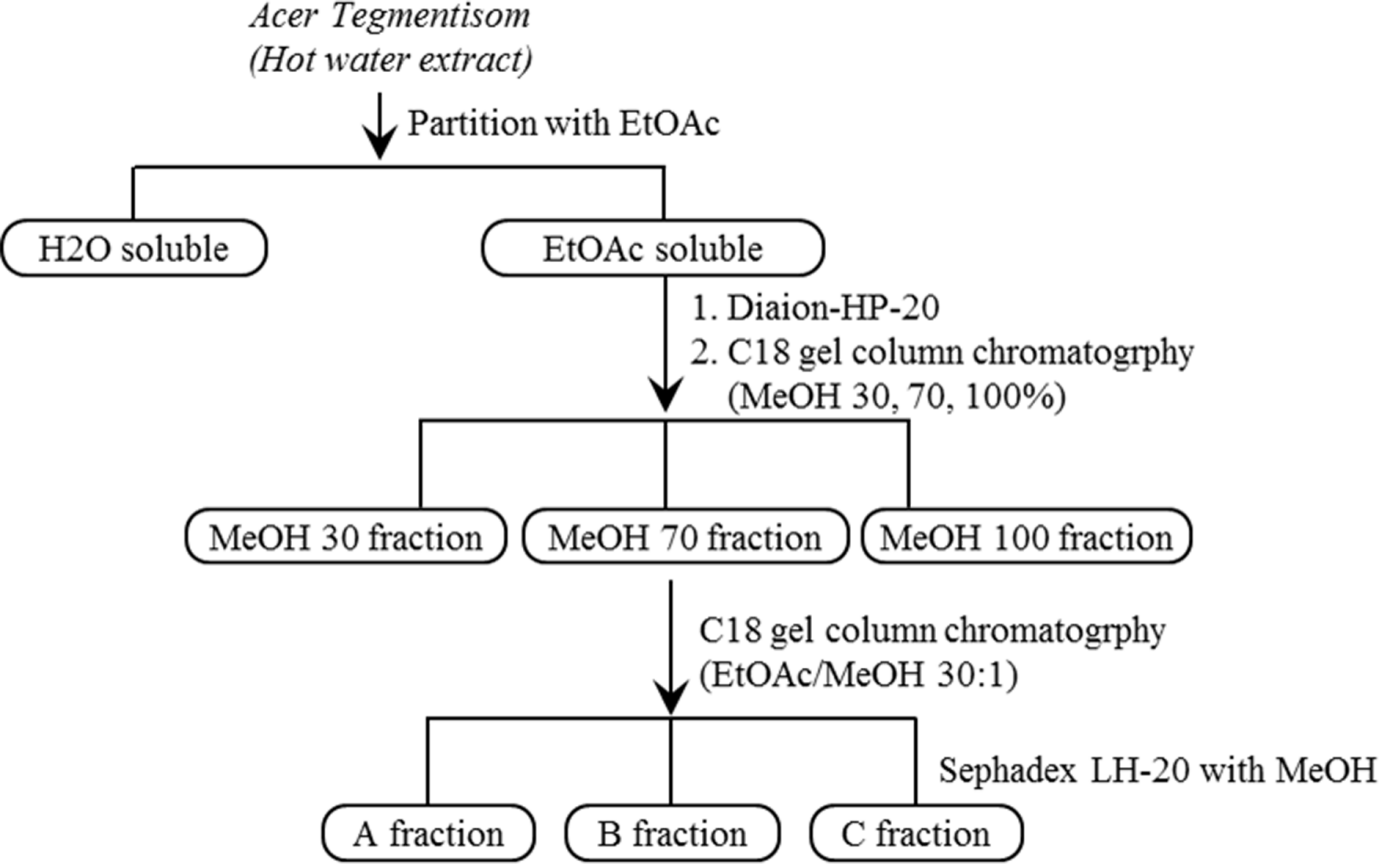
Active compound isolation and identification. A scheme of the chromatographic methods used for the separation of *A*. *tegmentosum’*s active compound.

The barks of *A*. *tegmentosum* were dried at 60°C for 24 h and then pulverized. The dried and pulverized *A*. *tegmentosum* barks (200 g) were then subjected to extraction using distilled water (1 L) at 90°C for 10 h and filtered. The filtrate was concentrated in vacuo to obtain the resulting extracts (13.2 g). The extracts (459 g) were then suspended in distilled water (700 mL) thrice and successively solvent-partitioned using EtOAc. The EtOAc-soluble fraction was loaded to a Diaion HP-20 chromatography column and fractionated with 500 mL in each solvent system of 20%, 40%, 60%, 80%, and 100% MeOH in H_2_O. According to TLC analysis, 80% and 100% MeOH-miscible fractions were combined into the one fraction. The combined fraction (4.2 g) was then separated on a RP-C18 silica gel (230–400 mesh) chromatography column and eluted using a gradient solvent system to obtain three fractions (MeOH30, 70, 100 (%)). Fraction MeOH70 (3.3 g) was subjected to a silica gel (230–400 mesh) chromatography column and separated using a gradient solvent system of EtOAc-MeOH (30:1–1:1, v/v) to further obtain three fractions (A–C). Fraction C (928 mg) was subjected to chromatography using a Sephadex LH-20 column and separated with 100% MeOH to obtain eight subfractions (C1–8). Subfraction C7 (22 mg) was further purified by utilizing semipreparative reversed-phase HPLC with an isocratic solvent system of 44% MeOH (flow rate: 2 mL/min) to acquire the active compound (1.2 mg, tR = 44.0 min) (Fig 1). The compound was speculated as a known compound and identified to be from *A*. *tegmentosum* for the first time (S1 and S2 Figs, S1 Table). This compound was named as HIMH0021.

### HIMH0021 inhibited TNFα release in chronic-plus-binge feeding mice

**Fig 2:**
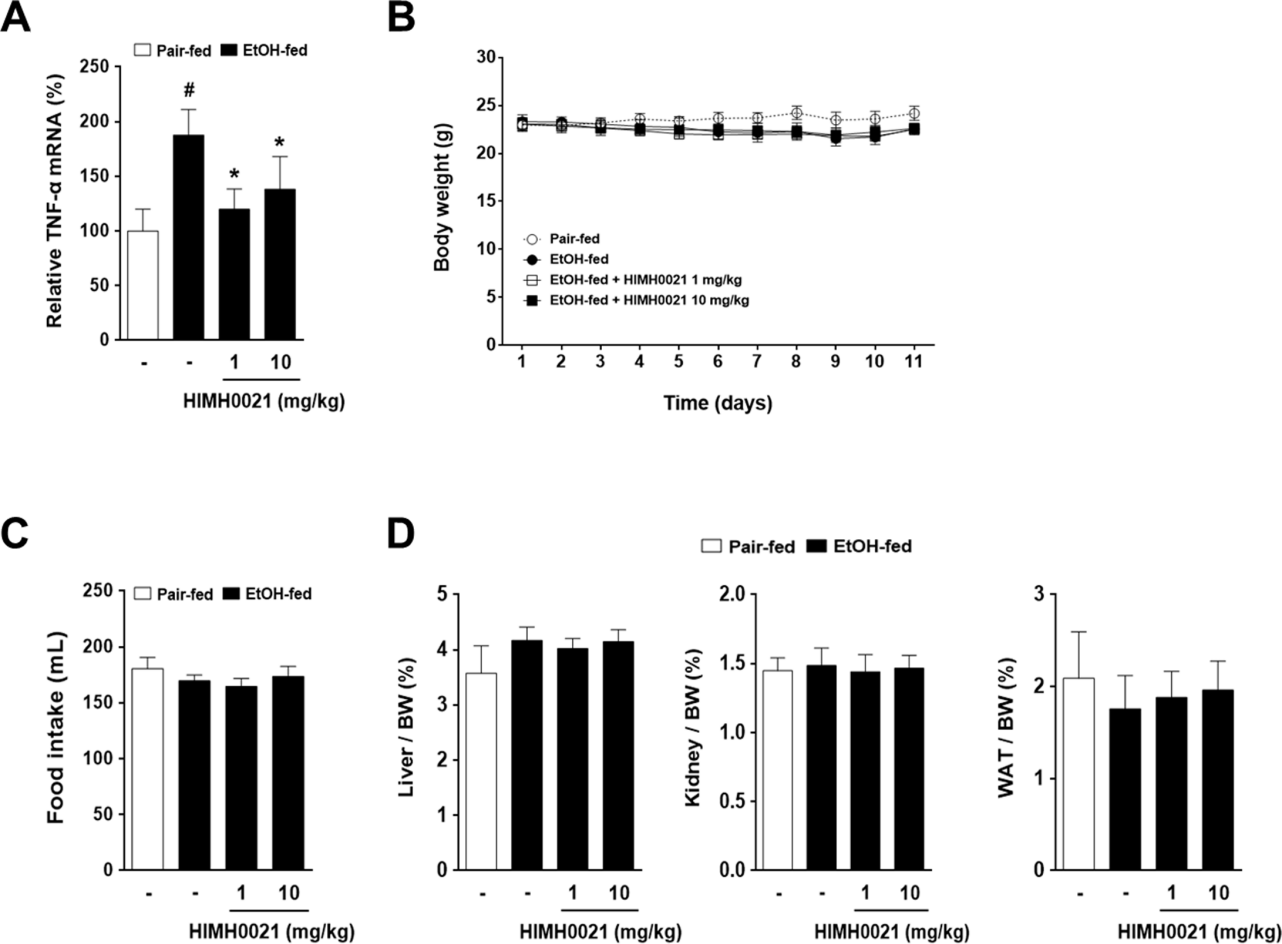
HIMH0021 inhibits tumor necrosis factor-α (TNF-α) release in chronic-plus-binge fed mice. (A) TNFα level was also assessed through real-time PCR using SYBR green in the liver samples from each treatment group at the end of the study. (B) Effect of HIMH0021 on body weight. (C) Effect of HIMH0021 on food intake. (D) Effect of HIMH0021 on the liver weight/body weight ratio, kidney weight/body weight ratio, and white adipose tissue weight/body weight ratio. The data shows mean ± standard deviation (SD) (n = 7). #p < 0.05, *p < 0.05, statistically significant difference.

Several studies have suggested that TNF-α mediates the early stages of fatty liver disease as well as the transition to more advanced stages of liver damage [21, 22]. Therefore, we examined whether HIMH0021 reduces the release of TNF-α by measuring the levels of TNF-α using RT-PCR. The alcohol-fed group showed a significant increase in the plasma levels of TNFα (Fig 2B). However, the increase in the mRNA levels of TNF-α was significantly inhibited by HIMH0021 treatment at doses of 1 and 10 mg/kg (Fig 2B). The alcohol-fed and alcohol + HIMH0021-fed groups showed a slight reduction in body weight and food intake compared to the pair-fed group (Fig 2C and 2D). However, there were no significant differences in the liver, kidney, and WAT between the pair-fed, alcohol-fed, and alcohol + HIMH0021-fed groups (Fig 2E).

### HIMH0021 treatment resulted in the recovery of adiponectin levels in chronic-plus-binge fed mice

**Fig 3:**
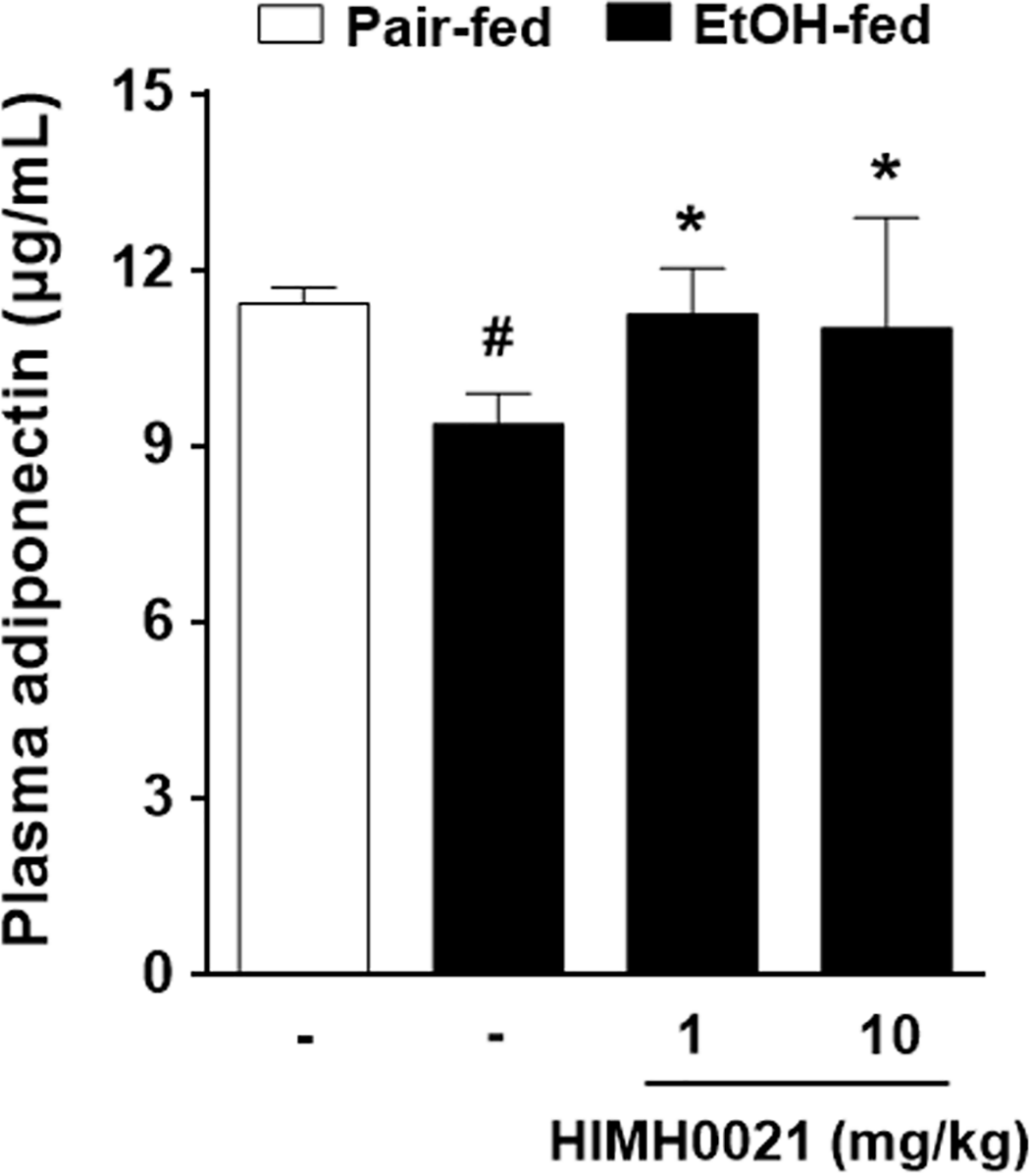
HIMH0021 treatment results in the recovery of adiponectin levels in chronic-plus-binge fed mice. Adiponectin levels were determined using ELISA in the plasma samples from each treatment group at the end of the study. The data shows mean ± SD (n = 7). #p < 0.05, *p < 0.05, statistically significant difference.

Adiponectin, a major anti-inflammatory factor, is an adipose tissue-derived adipokine. Adiponectin production is significantly impaired in patients with chronic ethanol exposure; down-regulation of adiponectin is also of pathophysiological importance in the process of alcoholic fatty liver disease (AFLD) development. To evaluate the role of HIMH0021 in the regulation of adiponectin, we analyzed the plasma levels of adiponectin using ELISA. As shown in Fig 3, plasma adiponectin levels were significantly decreased in the alcohol-fed group. In contrast, the plasma adiponectin levels in the alcohol + HIMH0021-fed group were higher, almost equal to that in the pair-fed group.

### HIMH0021 administration suppressed alcoholic hepatitis

**Fig 4:**
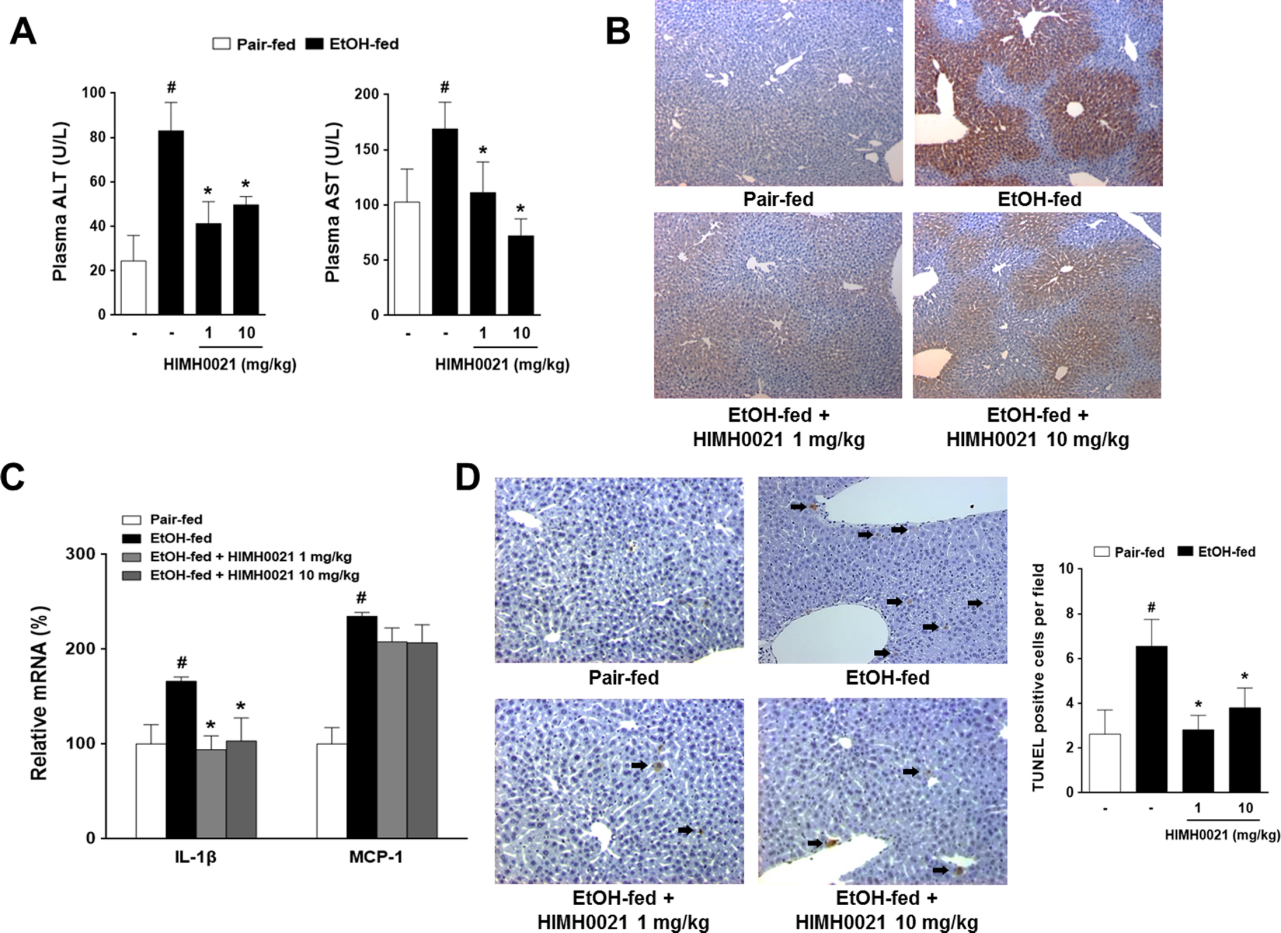
HIMH0021 suppresses alcoholic hepatitis. (A) Effects of HIMH0021 treatment on the plasma levels of ALT and AST. (B) Effect of HIMH0021 on CYP2E1 expression in liver tissues, as determined by immunohistochemistry (n = 7 per group). Original magnification, X 100. (C) Levels of IL-1β and MCP-1 in the liver samples were assessed by real-time PCR using SYBR green. (D) TUNEL assay was performed on the liver sections. Arrows denote apoptotic cells. Quantitation of TUNEL staining is shown. Apoptotic cells in 3 randomly selected fields were counted. The data is shown as mean ± SD (n = 7). #p < 0.05, *p < 0.05, statistically significant difference.

ALT and AST are the major markers of hepatic damage in the plasma. Therefore, in this study, we evaluated the plasma levels of ALT and AST in the pair-fed, alcohol-fed, and alcohol + HIMH0021-fed groups. The alcohol-fed group showed significantly higher levels of ALT and AST than the pair-fed group. However, the levels of ALT and AST were markedly decreased in the alcohol + HIMH0021-fed group (Fig 4A). As shown in Fig 4B, immunohistochemistry revealed the induction of CYP2E1 in the alcohol-fed group. However, the liver tissues of the mice in the alcohol- + HIMH0021-fed group displayed significantly weaker CYP2E1 staining compared to those of the alcohol-fed group. The monocyte chemoattractant protein 1 (MCP1), referred to as chemokine (C-C motif) ligand 2 (CCL2) is one of the key chemokine that regulate pathogenesis of several diseases characterized by monocytic infiltrates. Interleukin 1 beta (IL1β) also known as leukocytic endogenous mediator and lymphocyte activating factor, is a member of the interleukin 1 family of cytokines. This cytokine is produced by activated macrophages, which is an important mediator of the inflammatory response at tissue injury sites. In our study, we showed that the mRNA levels of MCP-1 and IL-1β were higher in the alcohol-fed group than in the pair-fed group; in contrast, the alcohol + HIMH0021-fed group showed decreased mRNA levels of both these factors (Fig 4C). Furthermore, apoptosis was analyzed in the liver tissues using the TUNEL assay. The number of apoptotic cells in the alcohol-fed group was higher than that in the pair-fed group (Fig 4D). However, the number of apoptotic cells was significantly decreased in the alcohol + HIMH0021-fed group compared to that in the alcohol-fed group.

### HIMH0021 suppressed hepatic lipid accumulation

**Fig 5:**
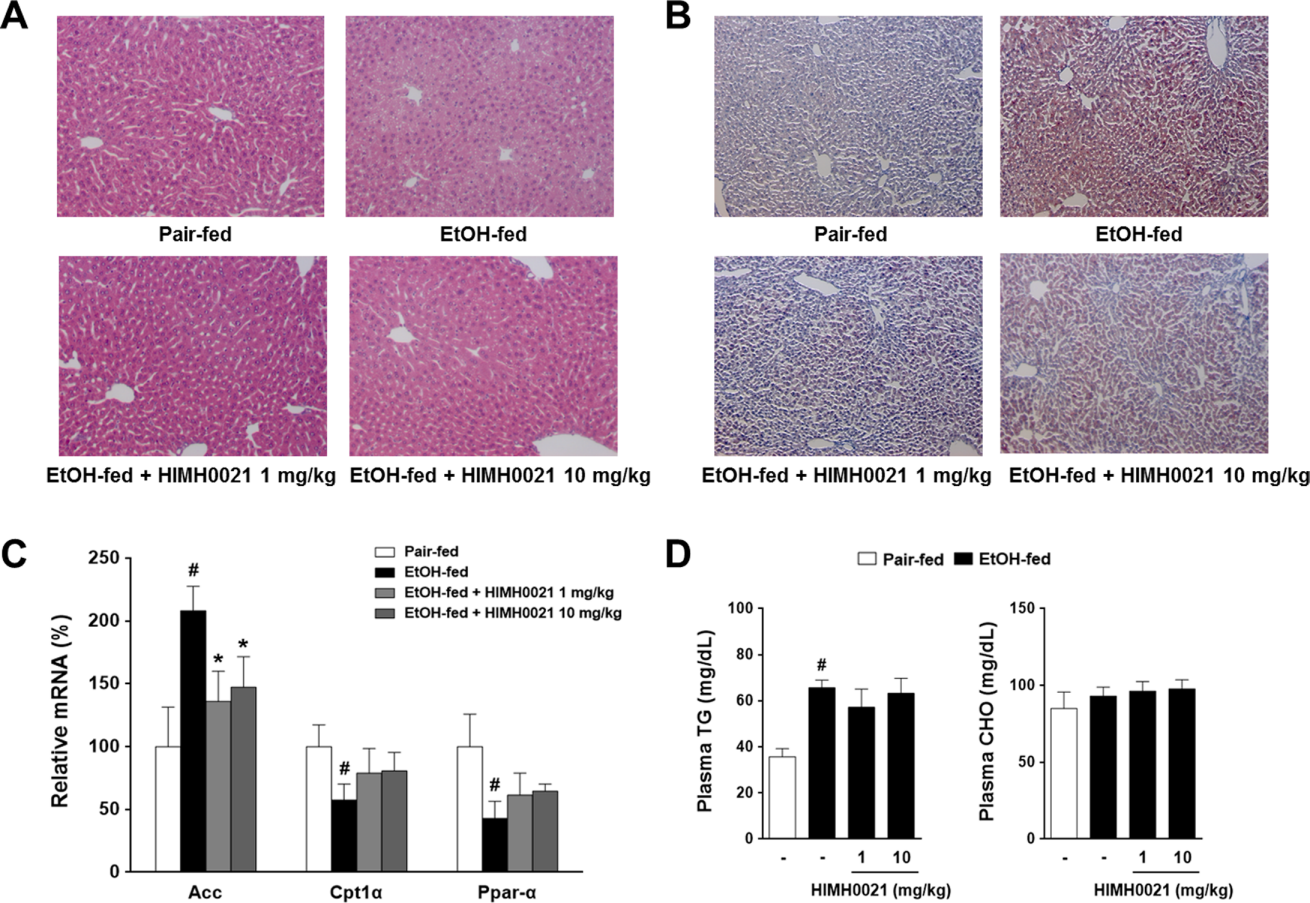
HIMH0021 suppresses hepatic lipid accumulation. (A) Representative photomicrographs of hematoxylin and eosin-stained liver sections. (B) The representative photomicrographs of Oil Red O-stained liver sections. (C) Quantitative real-time PCR analysis of genes involved in hepatic fatty acid synthesis. (D) Effect of HIMH0021 on plasma triglyceride and plasma cholesterol levels. The data is shown as mean ± SD (n = 7). #p < 0.05, *p < 0.05, statistically significant difference.

Histopathological analysis revealed that ethanol administration caused a higher degree of microvesicular and macrovesicular steatosis in the livers of alcohol-fed mice compared to that in the pair-fed mice (Fig 5A). However, the HIMH0021-treated mice showed reduced lipid accumulation and a substantial improvement in liver morphology compared to the alcohol-fed mice (Fig 5B). We also examined the expression of genes that regulate lipid metabolism to investigate whether HIMH0021 could inhibit alcohol-induced hepatic steatosis. Expression of the lipogenic gene, acetyl-CoA carboxylase (ACC), was significantly upregulated in the alcohol-fed mice; however, HIMH0021 treatment significantly decreases the mRNA level of ACC [P<0.05]. In contrast, the expression levels of carnitine palmitoyltransferase1 α (CPT1 α) and peroxisome proliferator-activated receptor α (PPAR α) were decreased in the alcohol-fed mice. HIMH0021 treatment slightly restored the expression levels of these genes (Fig 5C). In addition, slightly lower levels of serum TG were detected in the HIMH0021-fed mice than in the alcohol-fed mice, but this difference was not significant (Fig 5D). However, no difference was observed in the total cholesterol level in the sera between the different groups.

### HIMH0021 suppressed hepatitis through AMPK signaling

**Fig 6:**
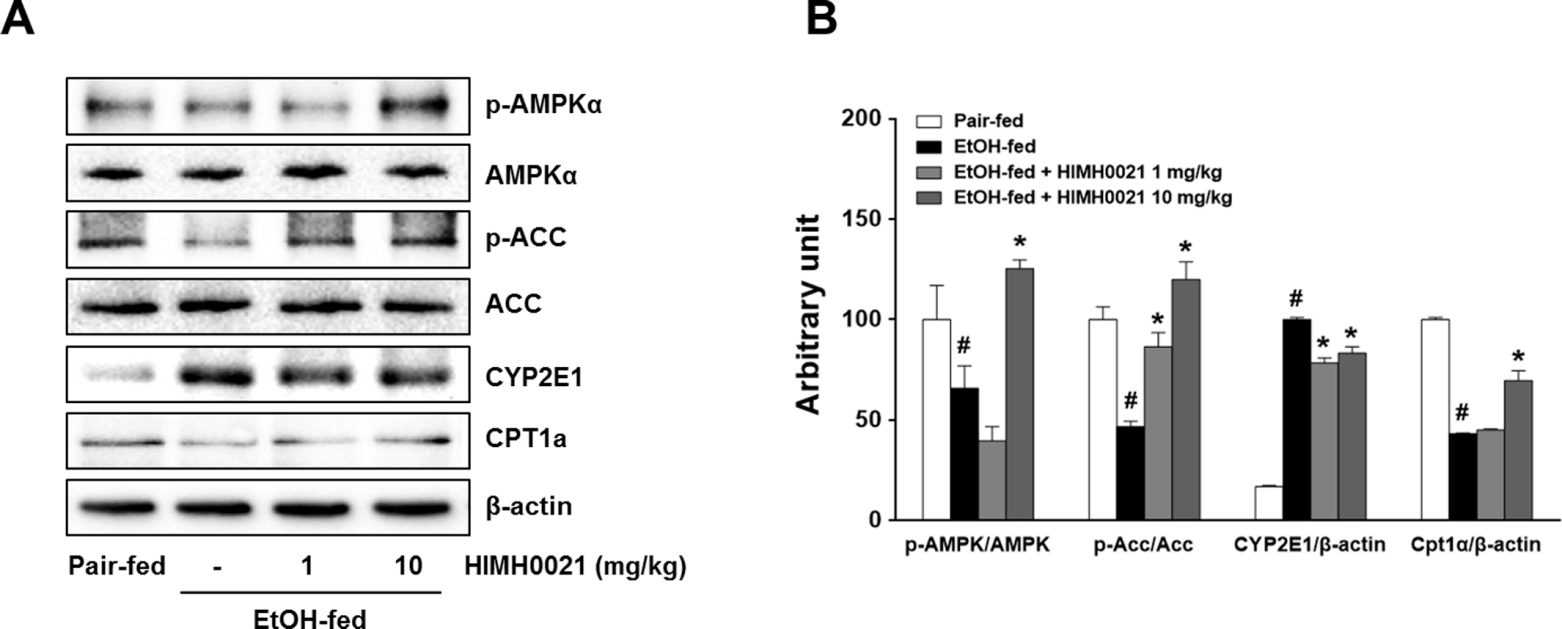
HIMH0021 induces hepatic AMPK activity in alcohol-induced hepatitis. (A) Western blot analysis of AMPK and ACC phosphorylation, CYP2E1, and CPT1 in mouse livers. (B) Relative protein band quantification was performed based on the optical density data from (A). The data is shown as mean ± SD (n = 7). #p < 0.05, *p < 0.05, statistically significant difference.

To understand the mechanisms involved in the pathogenesis of AFLD, we also analyzed the hepatic expression of p-AMPKα, p-ACC, CYP2E1, and CPT-1, which regulate hepatic fat metabolism. We found that the expression of these molecules was suppressed in the alcohol-fed mice, which promoted alcoholic fatty liver (except for CYP2E1). The AMPK protein levels were not different between the groups, but the p-AMPK levels were higher in the HIMH0021 (10 mg/kg)-treated group than in the other groups. The data indicated that HIMH0021 caused an increase in AMPK phosphorylation in a concentration-dependent manner. The alcohol-fed mice showed significantly higher hepatic CYP2E1 levels than the pair-fed mice, but supplementation with HIMH0021 reduced these levels. The expression of p-ACC and CPT-1 in the liver was also examined by western blot analysis. The alcohol-fed mice had significantly lower p-ACC and CPT-1 levels than the pair-fed mice. However, HIMH0021 significantly reversed this effect of ethanol on hepatic p-ACC and CPT-1 levels (Fig 6).

## Discussion

Alcohol-related disease is a liver inflammatory disease caused by alcohol-induced hepatic steatosis and liver injury caused by alcohol intake, and accounts for more than 10% of the cases of serious liver diseases in the world. Alcohol-related disease is a syndrome of progressive inflammatory liver injury associated with long-term heavy ethanol intake; however, its pathogenesis is not completely understood. Patients with alcohol-related disease present with subacute onset of hepatic injury, inflammatory infiltration and liver failure, and manifestations of portal hypertension [23, 24]. Several drugs have been used to treat alcoholic hepatitis. Corticosteroids such as prednisolone have been used for the treatment of severe alcoholic hepatitis for more than 40 years, and clinical trials have been conducted to determine the efficiency of these steroids. Many treatments for alcoholic liver disease, such as opiate antagonists, hemorheologic agents, chelating agents, hepatotropic hormones, antioxidants, or TNFα antagonists, have been considered controversial. Moreover, although such steroids and drugs are effective treatments, renal toxicity and other side effects hinder the long-term use of these drugs.

Several studies have shown that *A*. *tegmentosum* extract attenuates TG synthesis and lipid accumulation through the regulation of lipogenesis and FA oxidation in hepatocytes. The major active components of *A*. *tegmentosum* responsible for this pharmacological activity include flavonoid compounds such as pyranoflavonol, naringenin, and quercetin [25–28]. In this study, our results showed that the active flavonoid compound, HIMH0021, extracted from *A*. *tegmentosum* could inhibit hepatic lipogenesis as well as liver inflammation caused by alcohol consumption and thus protect the liver from alcohol-induced hepatic apoptosis and liver injury in mice.

The use of animal models represents a critical step in the process of informing plans for identifying the most promising candidate medications that can be advanced to clinical trials. During the last few decades, numerous animal models have been developed to study excessive levels of alcohol self-administration for screening candidate drugs. Recently, the chronic-binge EtOH feeding model, a rodent model with the more severe alcohol-induced liver injury model, has been suggested to reveal valuable insights into alcohol-related diseases such as chronic-binge EtOH feeding-induced severe hepatic steatosis, oxidative stress, and inflammation in mice. Here, we used this model to investigate the effect of HIMH0021 treatment on ALD. Moreover, to prevent hepatic pathology, we treated the mice with HIMH0021 to inhibit fatty liver development and the expression of the inflammatory cytokines, TNF-α and IL-1β, and that of CYP2E1 enzymes. Together, these results indicate the possibility that regulation of pAMPK activity by HIMH0021 in liver tissue modulates fatty acid oxidation, which further increases the lipid degradation level of hepatic cells and inhibits hepatitis. Based on the effect of HIMH0021 on alcoholic fatty liver and liver injury, future research will focus on the improvement of homeostatic lipid metabolism in the liver.

In summary, our present study shows, for the first time, that HIMH0021 ameliorates alcohol-induced fatty liver and liver injury by improving hepatic energy metabolism and preventing hepatic apoptosis. We also believe that the effect of HIMH0021 may be due to the activation of the AMPK signaling pathway. Thus, our study provides crucial information about the possible use of HIMH0021 as a clinical candidate for the prevention and treatment of alcohol-related diseases.

## Supporting information

S1 Fig ^1^H (700MHz) and ^13^C (175MHz) NMR spectrum of HIMH0021. (A) ^1^H (700MHz) and (B) ^13^C (175MHz) NMR spectrum.
(TIF)

S2 Fig ESI MS spectrum of HIMH0021. A negative ion-mode m/z 433 [M—H] -, positive mode m/z 435 [M+H] + and 457 [M+Na] +.
(TIF)

S1 Table ^1^H(700MHz) and ^13^C(175MHz) NMR data of HIMH0021 in CD_3_OD. (d in ppm).
(TIF)
