# Supplementary figures and images for: HIMH0021 attenuates ethanol-induced liver injury and steatosis in mice
Source: PLoS One. 2017 Nov 1;12(11):e0185134. doi: 10.1371/journal.pone.0185134 (PMC5665428; doi:10.1371/journal.pone.0185134)

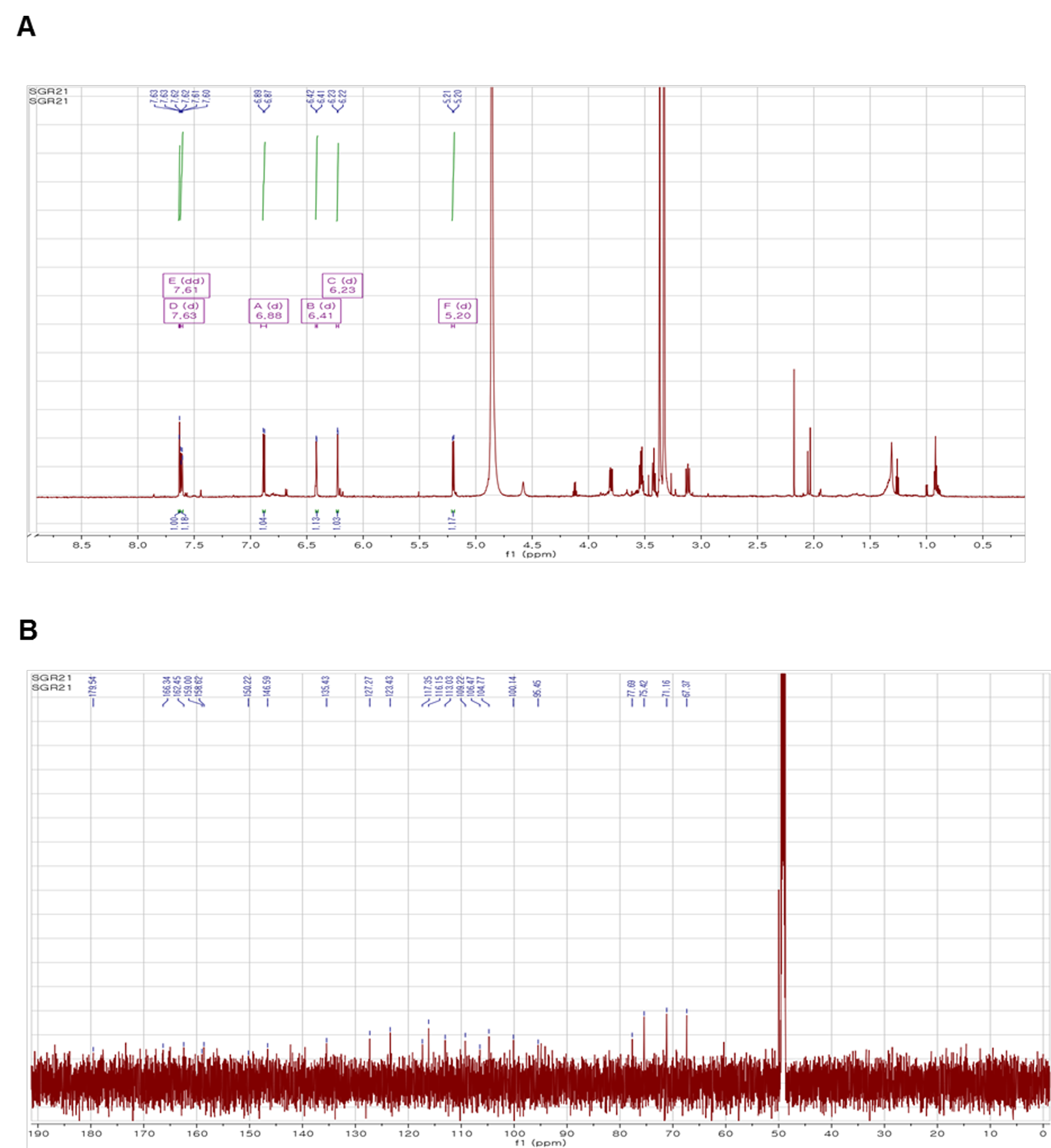

Supplement: S1 Fig — (A) 1H (700MHz) and (B) 13C (175MHz) NMR spectrum. (TIF) [file pone.0185134.s001.tif]

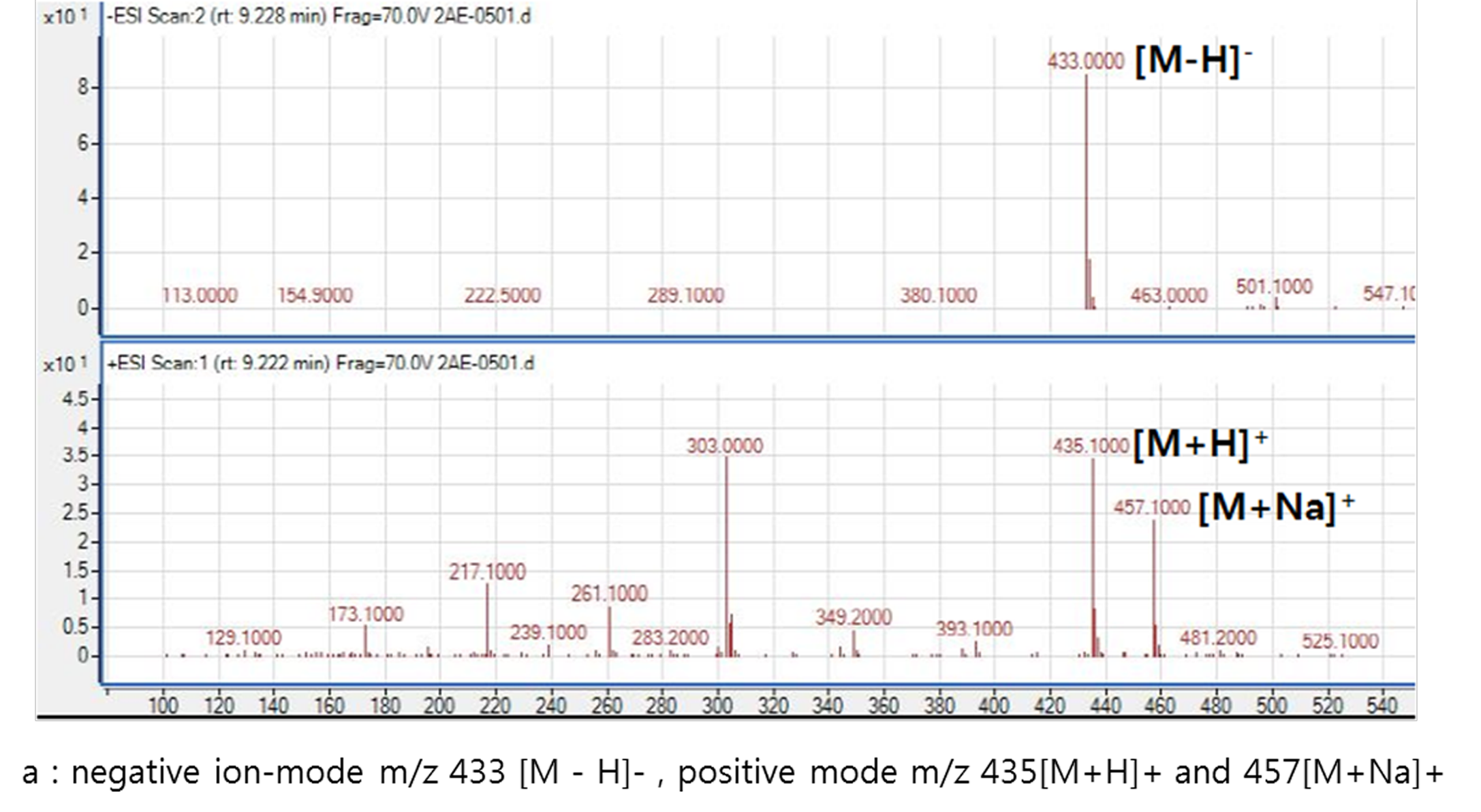

Supplement: S2 Fig — A negative ion-mode m/z 433 [M—H] -, positive mode m/z 435 [M+H] + and 457 [M+Na] +. (TIF) [file pone.0185134.s002.tif]

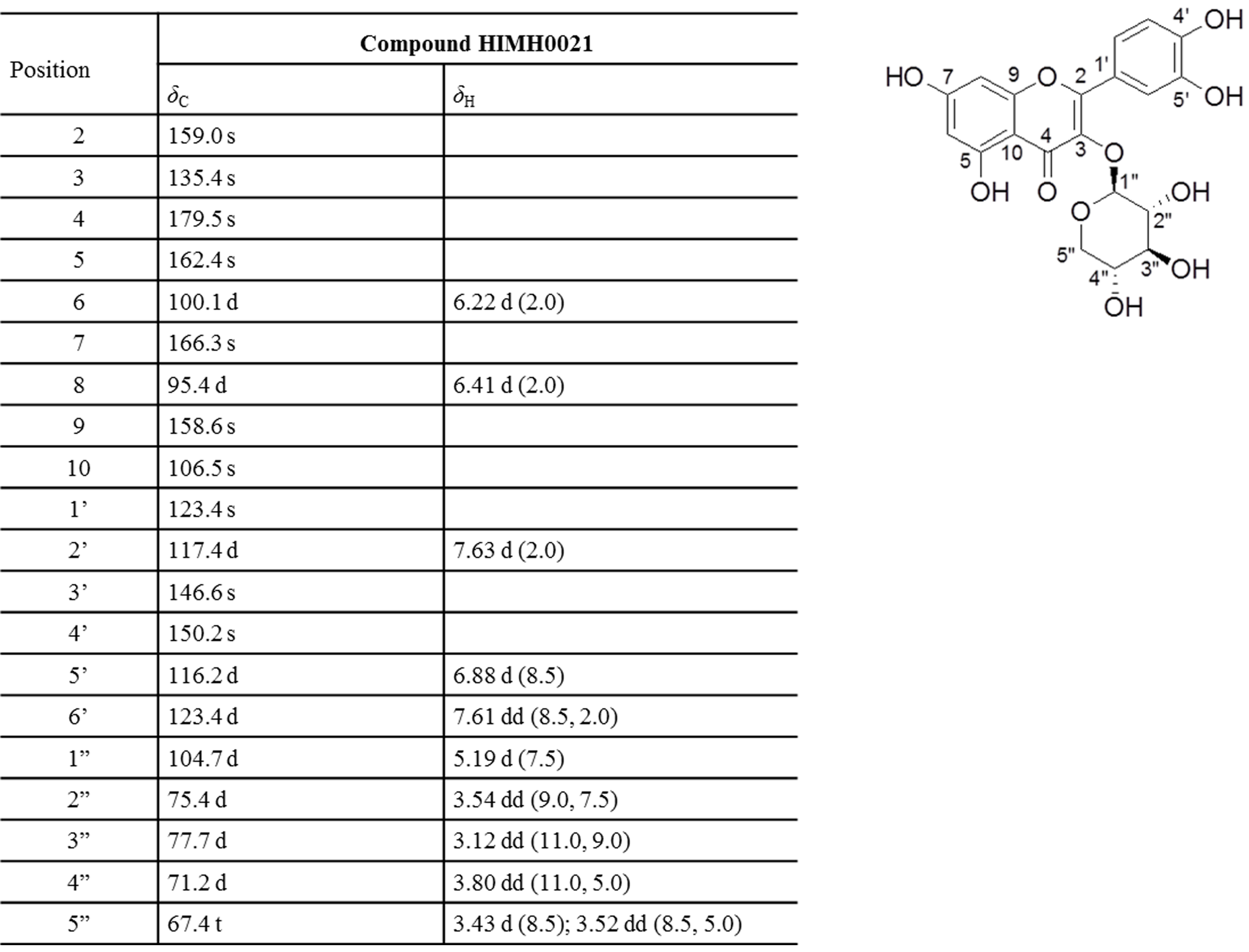

Supplement: S1 Table — (d in ppm). (TIF) [file pone.0185134.s003.tif]
